# Supplementary material for: Content-rich biological network constructed by mining PubMed abstracts
Source: BMC Bioinformatics. 2004 Oct 8;5:147. doi: 10.1186/1471-2105-5-147 (PMC528731; doi:10.1186/1471-2105-5-147)
Supplement: Additional File 2 — The original results of the above study (non-essential files are deleted to keep the file size under the limit set by BMC bioinformatics). [file 1471-2105-5-147-S2.bz2 › chilibotAdditionalFile2/dip30/704ID10734142E18041/html/MUTL_MUTS.html]

 


 **MUTL** and **MUTS** 
  
Found 186 abstracts in PubMed, retrieved 30.  
 

 What does Google say? 
 PDF only 
| .edu only 

---

**Interactive relationship** (e.g. stimulation, inhibition, etc)

**Neutral relationship**- Such a higher order  **MutS**  complex productively interacts with  **MutL**  in ATP hydrolyzing conditions and generates a specific ternary complex, which might communicate with MutH.  Ref: 11888282 Biochemistry, 2002
- Human mismatch repair protein  **MutL**  homologue 1 MLH1 interacts with Escherichia coli  **MutL**  and  **MutS**  in vivo and in vitro a simple genetic system to assay MLH1 function.  Ref: 12513688 Biochem J, 2003
- The pathway in eukaryotes consists of the DNA binding components, which are the homologs of the bacterial  **MutS**  protein MSH 2 6, and the  **MutL**  homologs, which bind to the  **MutS**  homologs and are essential for the repair process.  Ref: 12091911 Nat Genet, 2002
- Both pathways require the formation of a  **MutS**  homolog complex which interacts with a second heterodimer, composed of two  **MutL**  homologs.  Ref: 12836196 Med Sci Paris , 2003
- **MutS**  initiates repair by binding to the mismatch, and activates together with  **MutL**  the MutH endonuclease, which incises at hemimethylated dam sites and thereby mediates strand discrimination.  Ref: 11920679 J Cell Physiol, 2002
- **MutL**  related proteins were also required for mutation avoidance in stationary phase cells, but to a lesser extent than  **MutS**  homologues.  Ref: 12491007 Curr Genet, 2002

**Non-interactive relationship** (e.g. studied together, co-existance, homology, etc.)

- As compared to native  **MutS** , the ability of MutS800 to promote  **MutL**  dependent activation of MutH is substantially reduced.  Ref: 12829697 J Biol Chem< MedlineTA>J Biol Chem, 2002
- In eukaryotes, homologs of the Escherichia coli  **MutS**  and  **MutL**  proteins are crucial for both meiotic recombination and post replicative DNA mismatch repair.  Ref: 12836196 Med Sci Paris , 2003
- The average percentages of positively stained cells for human  **MutL**  homolog 1 hMLH1, human  **MutS**  homolog 2 hMSH2, and human  **MutS**  homolog 6 hMSH6 in RGP CMM 75.6 3.4%, 67.20 7.71%, and 76.6 2.1%, respectively were reduced compared with benign nevi.  Ref: 12112321 Mol Carcinog, 2002
- These effects parallel the  **MutL**  and  **MutS**  binding and ATP binding hydrolysis activities of the mutated proteins.  Ref: 12513688 Biochem J, 2003
- However, several  **MutS**  like and  **MutL**  like proteins also participate in meiotic recombination.  Ref: 12095912 Hum Mol Genet, 2002
- We also describe the alterations in the  **mutS** ,  **mutL**  and uvrD genes responsible for the mutator phenotype of hypermutable P. aeruginosa strains isolated from CF patients.  Ref: 11952911 Mol Microbiol, 2002
- Following a common mismatch recognition step effected by  **MutS**  homologous Msh proteins, homologs of the bacterial  **MutL**  ATPase predominantly the Mlh1p Pms1p heterodimer in yeast couple mismatch recognition to the appropriate downstream processing steps.  Ref: 12454061 Genetics, 2002
- Strand specificity and repair efficiency are largely independent of the GATC methylation state of the DNA and presence of the products of mismatch repair genes mutH,  **mutL** , and  **mutS** .  Ref: 12692132 J Biol Chem< MedlineTA>J Biol Chem, 2002
- MutH,  **MutL**  and  **MutS**  are essential components of the mismatch repair system in Escherichia coli.  Ref: 11934505 FEMS Microbiol Lett, 2002
- Protein components responsible for these reactions are highly conserved through evolution, and homologs of bacterial  **MutS**  and  **MutL** , which are key players in the initiation steps of both the strand specific mismatch correction and MMR dependent apoptotic signaling, have been identified in human cells.  Ref: 12700127 Front Biosci, 2003
- Exonuclease 1 Exo1 is a 5 3 exonuclease that interacts with  **MutS**  and  **MutL**  homologs and has been implicated in the excision step of DNA mismatch repair.  Ref: 12629043 Genes Dev, 2003
- The results shown here indicate that the putative P. aeruginosa  **mutS** ,  **mutL**  and uvrD genes are mutator genes.  Ref: 11952911 Mol Microbiol, 2002
- To address these questions, mice with inactivating mutations in all the known  **mutS**  and  **mutL**  homologs have been generated.  Ref: 12114115 Trends Mol Med, 2002
- RESULTS All cell lines examined showed decreased levels of  **MutS**  and  **MutL**  DNA MMR proteins in a dose dependent manner after coculture with H. pylori strains.  Ref: 12145807 Gastroenterology, 2002
- **MutS**  hMSH2 and hMSH6 and  **MutL**  hMLH1, hPMS2, and hPMS1 DNA MMR protein and RNA levels were determined.  Ref: 12145807 Gastroenterology, 2002
- Multiple  **MutS**  and  **MutL**  homologues exist in eukaryotes, which play different roles in the mismatch repair MMR pathway or in recombination.  Ref: 11920679 J Cell Physiol, 2002
- To investigate the effects on PV rates of mutations in genes for mismatch repair MMR, insertion deletion mutations of  **mutS** ,  **mutL** , mutH, dam, polI, uvrD, mfd and recA were constructed in Hi strain Rd.  Ref: 11889052 EMBO J, 2002
- At a low dose, dP is much more mutagenic in mismatch repair defective mutH,  **mutL** , and  **mutS**  strains than in a wild type strain.  Ref: 12196386 Genetics, 2002
- BACKGROUND The aim of this study was to search for mutations in the human  **mutS**  homolog 2 hMSH2 and human  **mutL**  homolog 1 hMLH1 genes in 25 unrelated Brazilian kindreds with suspected hereditary nonpolyposis colorectal cancer HNPCC.  Ref: 12095971 Ann Surg Oncol, 2002
- The mismatch repair MMR system plays a central role in maintaining genetic stability and requires evolutionarily conserved protein factors, including  **MutS**  and  **MutL**  homologs.  Ref: 12095912 Hum Mol Genet, 2002
- The human mismatch repair hMMR system consists of members of three super families, namely MutH,  **MutL**  and  **MutS** .  Ref: 12578652 Zhongguo Shi Yan Xue Ye Xue Za Zhi< MedlineTA>Zhongguo Shi Yan Xue Ye Xue Za Zhi, 2002
- Specific ternary complexes of  **MutS**   **MutL**  heteroduplexes were formed only during ATP hydrolysis.  Ref: 11888282 Biochemistry, 2002
- In vitro and in vivo studies of  **MutS** ,  **MutL**  and MutH mutants correlation of mismatch repair and DNA recombination.  Ref: 12606120 DNA Repair Amst , 2003
- The mutator phenotype was due to the ability of hMLH1 protein to interact with bacterial  **MutL**  and  **MutS**  proteins, thereby interfering with the formation of complexes between MMR proteins and mismatched DNA.  Ref: 12513688 Biochem J, 2003
- An unsolved problem in E. coli mismatch repair is how the  **MutS**   **MutL**  complex communicates positional information of a mismatch to MutH.  Ref: 11888282 Biochemistry, 2002
- In order to understand the role of the yeast mismatch repair MMR system in the mutagenesis of stationary phase cells, we studied the effects of deletions in genes encoding  **MutS**  and  **MutL**  related proteins on the reversion frequency of the lys2 Delta Bgl frameshift mutation.  Ref: 12491007 Curr Genet, 2002
- Seven out of the 11 mutator strains were found to be defective in the MMR system four  **mutS** , two  **mutL**  and one uvrD.  Ref: 11952911 Mol Microbiol, 2002
- Finally, every mutation that abolishes mismatch repair is deficient in blocking homeologous recombination, suggesting that mismatch repair and prevention of homeologous recombination use the same  **MutS**   **MutL**  complexes for signaling in E. coli.  Ref: 12606120 DNA Repair Amst , 2003
- Whereas  **mutS**  and  **mutL**  genes are found in most organisms, the mutH gene is limited to some proteobacteria.  Ref: 11934505 FEMS Microbiol Lett, 2002
- Since the discovery of a link between the malfunction of post replicative MMR and human cancers, a number of works have focused on the function of  **MutS**  and  **MutL**  homologs in the correction of replication errors.  Ref: 12095912 Hum Mol Genet, 2002
- The  **MutS**  homologous MSH and  **MutL**  homologous MLH proteins are highly conserved among all prokaryotes and eukaryotes.  Ref: 12582547 Theor Appl Genet< MedlineTA>Theor Appl Genet, 2002
- We have used the recently determined crystal structures of Escherichia coli E. coli  **MutS** ,  **MutL**  and MutH to guide construction of 47 amino acid substitutions in these proteins and analyzed their behavior in mismatch repair and recombination in vitro and in vivo.  Ref: 12606120 DNA Repair Amst , 2003
- In four cases three  **mutS**  and one  **mutL** , the genes contained frameshift mutations.  Ref: 11952911 Mol Microbiol, 2002
- In eukaryotic cells, DNA mismatch repair is initiated by a conserved family of  **MutS**  Msh and  **MutL**  Mlh homolog proteins.  Ref: 12529393 Mol Cell Biol, 2003
- VSP repair and MMR share two proteins,  **MutS**  and  **MutL** , and although the two repair pathways are not equally dependent on these proteins, their dual use creates a competition within the cells between the repair processes.  Ref: 12067333 Mol Microbiol, 2002
- The  **mutS**  and  **mutL**  genes, which are contiguous in the L. monocytogenes chromosome, were identified after in silico analysis.  Ref: 11994166 Mol Microbiol, 2002
- The mismatch repair system  **mutS** ,  **mutL**  and uvrD genes in Pseudomonas aeruginosa molecular characterization of naturally occurring mutants.  Ref: 11952911 Mol Microbiol, 2002
- The DNA repair genes  **mutL**  and  **mutS**  were separated by the prophage remnant SF370.4.  Ref: 12441069 Virology, 2002
- the cause of MMR deficiency in these cell lines is due to a defective  **MutS**  alpha or  **MutL**  alpha.  Ref: 12173046 Oncogene, 2002
- Evidence to support a role for the mismatch repair genes human  **mutL**  homolog 1 hMLH1 and human  **mutS**  homolog 2 hMSH2 in the etiology of colorectal cancer has come from linkage analysis, segregation studies, and molecular biologic analysis.  Ref: 12419761 Am J Epidemiol, 2002
- The mammalian DNA mismatch repair genes belong to a family of genes that comprise several homologs of the Escherichia coli  **mutS**  and  **mutL**  genes.  Ref: 12114115 Trends Mol Med, 2002
